# Ciprofloxacin Concentrations 100-Fold Lower than the MIC Can Select for Ciprofloxacin Resistance in *Neisseria subflava*: An In Vitro Study

**DOI:** 10.3390/antibiotics13060560

**Published:** 2024-06-14

**Authors:** Zina Gestels, Saïd Abdellati, Chris Kenyon, Sheeba Santhini Manoharan-Basil

**Affiliations:** 1 Sexually Transmitted Infections Unit, Department of Clinical Sciences, Institute of Tropical Medicine, 2000 Antwerp, Belgium; ckenyon@itg.be (C.K.); sbasil@itg.be (S.S.M.-B.); 2 Clinical and Reference Laboratory, Department of Clinical Sciences, Institute of Tropical Medicine, 2000 Antwerp, Belgium; 3 Division of Infectious Diseases and HIV Medicine, University of Cape Town, Cape Town 7700, South Africa

**Keywords:** minimum selection concentration, MSC, MSC_de novo_, ciprofloxacin, *Neisseria subflava*, commensals, antimicrobial resistance

## Abstract

*Neisseria gonorrhoeae* can acquire antimicrobial resistance (AMR) through horizontal gene transfer (HGT) from other *Neisseria* spp. such as commensals like *Neisseria subflava*. Low doses of antimicrobials in food could select for AMR in *N. subflava,* which could then be transferred to *N. gonorrhoeae*. In this study, we aimed to determine the lowest concentration of ciprofloxacin that can induce ciprofloxacin resistance (minimum selection concentration—MSC) in a *N. subflava* isolate (ID-Co000790/2, a clinical isolate collected from a previous community study conducted at ITM). In this study, *Neisseria subflava* was serially passaged on gonococcal (GC) medium agar plates containing ciprofloxacin concentrations ranging from 1:100 to 1:10,000 below its ciprofloxacin MIC (0.006 µg/mL) for 6 days. After 6 days of serial passaging at ciprofloxacin concentrations of 1/100th of the MIC, 24 colonies emerged on the plate containing 0.06 µg/mL ciprofloxacin, which corresponds to the EUCAST breakpoint for *N. gonorrhoeae*. Their ciprofloxacin MICs were between 0.19 to 0.25 µg/mL, and whole genome sequencing revealed a missense mutation T91I in the *gyrA* gene, which has previously been found to cause reduced susceptibility to fluoroquinolones. The *N. subflava* MSC_de novo_ was determined to be 0.06 ng/mL (0.00006 µg/mL), which is 100×-fold lower than the ciprofloxacin MIC. The implications of this finding are that the low concentrations of fluoroquinolones found in certain environmental samples, such as soil, river water, and even the food we eat, may be able to select for ciprofloxacin resistance in *N. subflava.*

## 1. Introduction

The threat of antimicrobial resistance (AMR) is compromising the treatment of common infections, including sexually transmitted infections (STIs) such as gonorrhoea [1]. A key measure in studying AMR is the minimum inhibitory concentration (MIC) that is routinely used to measure the lowest concentration of an antibiotic that inhibits the growth of a microorganism. However, the selection of resistant bacteria is not limited to concentrations between the MIC of the susceptible wild-type population and that of the resistant bacteria [2]. De novo resistance development occurs not only above the MIC of the susceptible strain, but also at concentrations lower than the MIC, which can drive the selection of resistant mutants through mechanisms such as horizontal gene transfer (HGT) and mutations emphasizing the importance of the minimal concentration (MSC). The MSC is the lowest concentration of an antimicrobial that can select for antimicrobial resistance in a bacterium [2,3]. The MSC encompasses two components. The MSC_select_ denotes the lowest concentration that provides a selection pressure for resistant mutants over the susceptible strains. At this concentration, the fitness cost of resistance will allow the susceptible bacteria to out-compete the resistant bacteria, and the MSC_de novo_ is defined as the lowest concentration that can induce de novo AMR [2,3].

Studies have shown that sub-inhibitory concentrations of antimicrobials can create a selective environment that can result in the emergence of resistance. For example, Gullberg et al. established the ciprofloxacin MSC_select_ and MSC_de novo_ for *Escherichia coli* as 0.1 ng/mL and 2.3 ng/mL (1/230th and 1/10th the MIC), respectively. However, lower concentrations were not accessed; for instance, they did not assess if ciprofloxacin concentrations below 2.3 ng/mL could induce de novo resistance [2]. Previous MSC experiments with *Neisseria gonorrhoeae* revealed that ciprofloxacin concentrations as low as 0.004 ng/mL, or 1/1000th of the MIC, could induce de novo resistance [4]. Once again, lower concentrations were not tested in this study [4]. These MSCs are considerably lower than the maximum residue limits of fluoroquinolones allowed in various meat products by the European Medicines Authority and the Food and Agriculture Organization [4,5]. These MSCs are also orders of magnitude lower than the concentrations of ciprofloxacin detected in samples of milk, eggs, and edible fish in certain East Asian countries (mean concentration: 8.5 µg/L, 16.8 µg/kg and 331.7 µg/kg, respectively) [6,7,8]. Of further concern is that these MSCs are higher than the ciprofloxacin concentration detected in the faeces of random individuals in three regions of China (median concentration 20 μg/kg) [9]. This suggest the ingestion of veterinary antimicrobials in food could be responsible [10,11,12]. Additionally, low concentrations of antimicrobials in the soil and water may also select for AMR, potentially transferring resistance to humans or other animals. A global survey of pharmaceuticals in the world’s rivers found that the concentration of ciprofloxacin exceeded ‘safe levels’ of 60 ng/L at 64 out of 135 sites [13]. These country-level ciprofloxacin concentrations in rivers were found to be positively associated with the prevalence of fluoroquinolone resistance in *E. coli* [14].

These considerations mean it is important to establish the MSCs of a wider range of bacteria. In this study, we investigate the MSC_de novo_ for ciprofloxacin in *Neisseria subflava*, a commensal bacterium that is part of our normal oropharyngeal microbiota and can transfer DNA encoding antimicrobial resistance to the pathogenic *Neisseria* species, *N. gonorrhoeae* and *N. meningitidis* [15,16,17,18,19,20]. Commensal *Neisseria* have been found in the resident microbiomes of various food animals, including chickens, cows, sheep, and goats [21,22,23,24]. The selection of quinolone resistance in commensal *Neisseria* can therefore occur in both animals and humans. A number of studies have confirmed that horizontal gene transfer from commensal *Neisseria* spp. has played a crucial role in the emergence of resistance to fluoroquinolones, cephalosporins, dihydrofolate reductase inhibitors, and macrolides in *N. gonorrhoeae/N. meningitidis* [15,16,17,18,19,20].

A systematic review of AMR in *Neisseria* spp. found that resistance was typically higher in commensal compared to pathogenic *Neisseria* spp. [25]. This is likely related to the fact that the prevalence of the commensal *Neisseria* spp. is close to 100%, whereas that of the pathogenic *Neisseria* spp. is one or two orders of magnitude lower [26,27]. This higher prevalence means that the commensal *Neisseria* are exposed to antimicrobial selection pressure every time someone ingests an antimicrobial [27]. This may also mean that the commensal *Neisseria* are more susceptible to the effects of chronic low-dose exposure to fluoroquinolones, such as those in food [26]. This hypothesis is, however, dependent on the concentration of fluoroquinolones in foodstuffs being higher than the MSCs.

In the present study, we determined the *N. subflava* ciprofloxacin MSC_de novo_ by passaging *N. subflava* in ciprofloxacin concentrations ranging from 1:100 to 1:10,000 below the MIC for 6 days.

## 2. Results

### 2.1. Minimal Selective Concentration

#### 2.1.1. *N. subflava*

After 6 days of serial passaging at ciprofloxacin concentrations 1/100th of the MIC, equivalent to 0.00006 µg/mL, 24 colonies emerged after 22 h of incubation on a single 0.06 µg/mL ciprofloxacin plate (Plate 1/100-4; Table 1). MALDI-TOF MS analysis verified that these colonies were *N. subflava*.

E-testing of these colonies revealed a MIC of 0.19 to 0.25 µg/mL for all the colonies, which represents a minimal 31-fold increase in ciprofloxacin MIC. No colonies with resistance (0.06 µg/mL) were observed on the control or other plates passaged at 1/100, 1/1000, and 1/10,000 of the ciprofloxacin MIC.

#### 2.1.2. Whole Genome Sequencing: Mutations in Fluoroquinolone Target Gene (*gyrA*)

WGS analysis of five randomly selected isolates that grew on the ciprofloxacin plate, with a MIC ranging from 0.19 to 0.25 µg/mL, revealed a missense mutation T91I in the *gyrA* gene, the known resistant-associated mutation. Additionally, all four isolates had the missense mutation A385V in the *spoT* gene, which encodes the bifunctional (p)ppGpp synthase/hydrolase), and a synonymous mutation T828C (A276) in the *nnr* gene, which encodes a bifunctional NAD(P)H-hydrate repair enzyme).

### 2.2. Mutation Stability

Cross-plating of two strains (1/100-4.1 and 1/100-4.7) on GC agar + 1% IV was performed every 24 h for 6 days. E-testing at day 6 revealed an unchanged ciprofloxacin MIC for 1/100-4.7, and a slightly higher MIC for 1/100-4.1—from 0.19 µg/mL to 0.25 µg/mL.

## 3. Discussion

Exposure to low ciprofloxacin concentrations (0.06 ng/mL, equivalent to 0.00006 µg/mL) that were 100-fold lower than the MIC for six days resulted in the emergence of fluoroquinolone resistance in *N. subflava*. This resistance was associated with T91I substitution in GyrA. This mutation has been shown to be associated with an intermediate fluoroquinolone resistance phenotype in *N. meningitidis* [28]. Using similar methodologies, Gonzalez et al. found that exposure to lower ciprofloxacin concentrations (0.004 ng/mL) or 1000-fold lower than the MIC could induce de novo ciprofloxacin resistance in *N. gonorrhoeae* [4]. In contrast, Gullberg et al. found that the ciprofloxacin MSC_de novo_ in *E. coli* was higher (2.3 ng/mL), although lower concentrations were not tested [2]. These findings suggest that concentrations of ciprofloxacin as low as 0.004 ng/mL can select for ciprofloxacin resistance.

This finding suggests the need to reconsider the definition of ‘safe’ concentrations of fluoroquinolones in environmental and food samples. For example, in their global survey of the world’s rivers, Wilkinson et al. found alarming levels of pharmaceutical pollution [13]. One of their concerning findings was that the concentration of ciprofloxacin exceeded ‘safe’ levels of 0.06 ng/mL at 64 sites. This threshold of 0.06 ng/mL was determined by Bengtsson-Palme et al. by ascertaining what the lowest 1% minimum inhibitory concentration (MIC) was for a range of bacteria with available susceptibility data in the EUCAST dataset [29]. To adjust for the fact that the MSC may be an order of magnitude lower than the MIC, Bengtsson-Palme et al. set the safe concentration of ciprofloxacin at 10-fold lower than the lowest 1% MIC. The MSCs of *Neisseria* spp. are, however, 100- to 1000-fold lower than their MICs. Applying a 10-fold safety factor to these MSCs would mean that the safe concentrations of ciprofloxacin could not be 10-fold, but up to 10,000-fold lower than the lowest 1% MIC. While this hypothesis will require experimental validation, it does suggest that measured concentrations of ciprofloxacin in a much larger proportion of the world’s rivers may be selecting for AMR.

We have only considered the ciprofloxacin MSC_de novo_ of a single strain of *N. subflava* in a very simple in vitro model. All the resistant isolates emerged on a single agar plate. The in vitro MSC_select_ is typically lower than the MSC_de novo_ [2]. MSCs will likely be different in complex environmental and microbial matrices. For example, MSCs may be lower in polymicrobial communities [30]. On the other hand, the presence of other compounds, such as heavy metals and selective serotonin receptor inhibitors, can reduce the MSC [31]. Our experiment only ran for 6 days. We cannot exclude the possibility that longer exposures may have resulted in a lower ciprofloxacin MSC.

These limitations mean that further experiments are required to determine MSCs in complex environments such as in vivo. Only a single study has assessed the MSC in vivo. This study found that single doses of the lowest dose of ciprofloxacin concentration tested (0.6 ng/g) could induce ciprofloxacin resistance in *Klebsiella pneumoniae* [32]. This finding is concerning, as this concentration was 10-fold lower than the ciprofloxacin food concentration classified as safe by the Food and Agriculture Organization [32]. As reviewed in the Introduction, this concentration is also considerably lower than that of fluoroquinolones detected in foodstuffs in various countries [6,7,8,9,10,11,12,13].

A recent study estimated that AMR infections are responsible for between 1 and 5 million deaths per year [33]. Combating AMR requires a one-health approach, whereby all antimicrobial exposures are kept within safe thresholds [34].

## 4. Materials and Methods

### 4.1. Bacterial Strain

We used *N. subflava* Co000790/2, a clinical isolate collected in a previous community study performed at ITM [35]. This strain has a ciprofloxacin MIC of 0.006 µg/mL, as ascertained with E-testing in triplicate.

### 4.2. MSC_de novo_ Determination

The MSC_de novo_ of *N. subflava* Co000790/2 was ascertained via exposure to a constant concentration of ciprofloxacin at 1:100, 1:1000, and 1:10,000 of its ciprofloxacin MIC on GC agar plates (Difco GC medium, Becton Dickinson, Franklin Lakes, NJ, USA) with 1% isovitalex enrichment (Becton Dickinson) in 5% CO_2_ incubator at 36 °C. Control experiments were conducted simultaneously under identical conditions, except that the GC agar plates did not contain ciprofloxacin. The experiments were conducted in quadruplicate. Every 24 to 48 h, each lineage was passaged to a new plate with the same conditions by transferring a 1/4th loopful (Copan, Singapore, 10 µL loop) to the next plate. This process was continued for 6 days.

On day 7, the number of colonies of each lineage with reduced susceptibility to ciprofloxacin was established as follows: 100 µL of Phosphate Buffered Saline (PBS) solution containing the lawn of colonies (1.0 McFarland) from each plate (n = 4, per condition) was plated onto GC agar plates with no ciprofloxacin (n = 4 per condition) or with 0.06 µg/mL ciprofloxacin (n = 4 per condition), resulting in 8 plates per condition in total. The concentration of the ciprofloxacin plates (0.06 µg/mL) corresponds to the EUCAST breakpoint for *N. gonorrhoeae* [36]. The number of colonies was counted after 24 h of incubation at 36 °C. The lowest ciprofloxacin concentration with growth in the 0.06 µg/mL plates was defined as the MSC_de novo_.

### 4.3. Characterization of Colonies That Grew on Ciprofloxacin-Containing Plates

The MICs of colonies that grew on the ciprofloxacin plates were ascertained using gradient diffusion strips (Etest^TM^, bioMérieux, Craponne, France), following EUCAST guidelines. The species identity of these colonies was confirmed via MALDI-TOF (Bruker, Billerica, MA, USA).

### 4.4. Mutation Stability Assessment

Two resistant colonies (ID: 1/100-4.1 and ID: 1/100-4.7) were randomly selected from the plates containing 0.06 µg/mL ciprofloxacin for further experimentation to determine the stability of acquired mutations. Each strain was retrieved from frozen skimmed milk stored at −80 °C, replated on GC agar + 1% IV, and subcultured every 24 h for 6 days. Finally, the cultures were subjected to E-testing following EUCAST guidelines.

### 4.5. Whole Genome Sequencing

Five isolates (1/100-4.1, 1/100-4.7, 1/100-4.14, 1/100-4.21, and 1/100-4.24) and one isolate from day 5 of the control experiment exposed to no ciprofloxacin were outsourced for DNA isolation, library preparation, and whole genome sequencing (WGS) to Eurofins, Hamburg, Germany. Post-DNA-extraction, libraries were prepared using the TruSeq DNA library kit (Illumina Inc., San Diego, CA, USA), and multiplexing was performed using the Nextera DNA library kit (Illumina Inc., San Diego, CA, USA). Sequencing was carried out on the NextSeq6000 v2 platform (Illumina Inc.), generating 2 × 150 bp reads. Quality assessment of the raw reads was performed using FASTQC v0.11.9 [37]. The raw reads were then trimmed for quality (Phred ≥ 30) and length (≥32 bases) using Trimmomatic (v0.39) [38]. The processed reads were assembled with Shovill (v1.0.4) [39], which uses SPAdes for the de novo assembly (v3.14.0) [40] using the following parameters: —trim—depth 150—opts—isolate. The quality of the assembled de novo contigswas evaluated using Quast (v5.0.2) [41]. Genome annotation of the draft genome was carried out using Prokka (v1.14.6) [42]. The quality-controlled reads were mapped to the reference draft genome (Ns_Ctrl) using Snippy (https://github.com/tseemann/snippy, accessed on 3 March 2024). Single nucleotide polymorphisms (SNPs) were determined using default parameters. The raw reads are deposited at PRJNA1107029.

**Figure 1:**
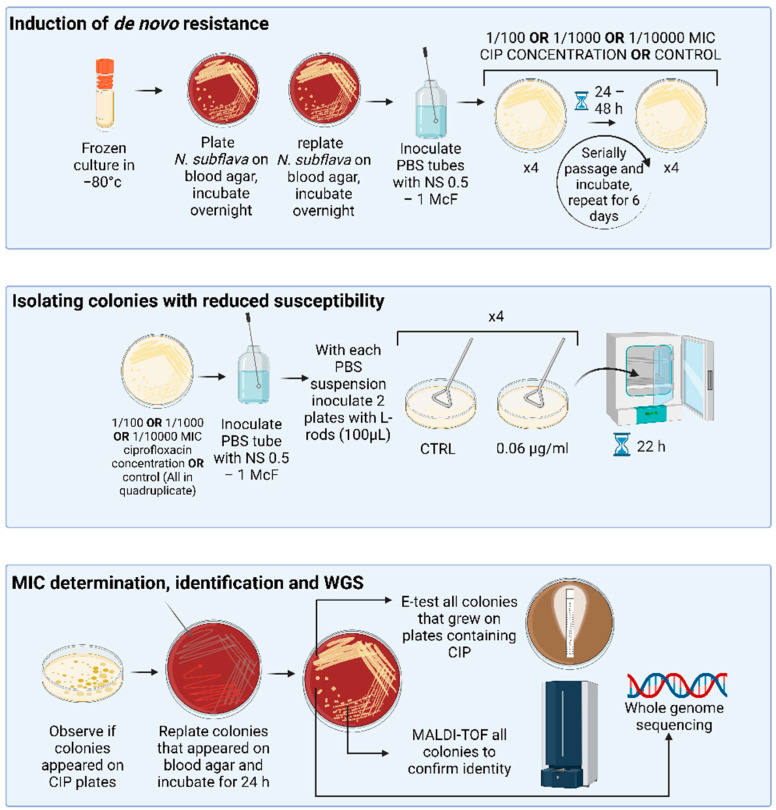
Overview of the study methodology, made with biorender.com.

Overview of the study is provided in Figure 1.

## 5. Conclusions

This study demonstrates that exposure to ciprofloxacin concentrations significantly lower than the minimum inhibitory concentration (MIC) can lead to the emergence of fluoroquinolone resistance in *Neisseria subflava*. Specifically, a T91I substitution in GyrA was associated with resistance (MIC 0.19 to 0.25 µg/mL). These results align with similar studies on *Neisseria gonorrhoeae* and *Escherichia coli*, suggesting that ciprofloxacin concentrations as low as 0.004 ng/mL are capable of selecting for resistance.

Given the presence of such low concentrations of ciprofloxacin in environmental samples, as highlighted by the global survey of pharmaceutical pollution in rivers by Wilkinson et al., there is a pressing need to reconsider the definition of ‘safe’ concentrations of fluoroquinolones [13]. The current thresholds may not adequately protect against the selection of antimicrobial resistance (AMR).

## Figures and Tables

**Table 1:** Minimum inhibitory concentrations of all resistant colonies, and subsequent MALDI-TOF results.

Colony	Ciprofloxacin MIC (µg/mL)	MALDI-TOF-MS ID	MALDI-TOF Score	Whole Genome Sequencing
1/100-4.1	0.19	*N. flavescens subflava* group	2.16	✓
1/100-4.2	0.19	*N. flavescens subflava* group	2.17	x
1/100-4.3	0.19	*N. flavescens subflava* group	2.26	x
1/100-4.4	0.19	*N. flavescens subflava* group	2.17	x
1/100-4.5	0.25	*N. flavescens subflava* group	2.21	x
1/100-4.6	0.19	*N. flavescens subflava* group	2.06	x
1/100-4.7	0.25	*N. flavescens subflava* group	2.01	✓
1/100-4.8	0.19	*N. flavescens subflava* group	2.12	x
1/100-4.9	0.19	*N. flavescens subflava* group	2.27	x
1/100-4.10	0.19	*N. flavescens subflava* group	2.3	x
1/100-4.11	0.19	*N. flavescens subflava* group	2.28	x
1/100-4.12	0.19	*N. flavescens subflava* group	2.19	x
1/100-4.13	0.19	*N. flavescens subflava* group	2.27	x
1/100-4.14	0.19	*N. flavescens subflava* group	2.25	✓
1/100-4.15	0.25	*N. flavescens subflava* group	2.07	x
1/100-4.16	0.25	*N. flavescens subflava* group	2.05	x
1/100-4.17	0.25	*N. flavescens subflava* group	2.11	x
1/100-4.18	0.25	*N. flavescens subflava* group	2.28	x
1/100-4.19	0.19	*N. flavescens subflava* group	2.28	x
1/100-4.20	0.25	*N. flavescens subflava* group	2.32	x
1/100-4.21	0.19	*N. flavescens subflava* group	2.31	✓
1/100-4.22	0.19	*N. flavescens subflava* group	2.25	x
1/100-4.23	0.19	*N. flavescens subflava* group	2.13	x
1/100-4.24	0.25	*N. flavescens subflava* group	2.27	✓

✓—Sequenced. x—Not sequenced.

## Data Availability

The data presented in this study are openly available in Genbank at https://www.ncbi.nlm.nih.gov/bioproject/PRJNA1107029/, (accessed on 23 May 2024), BioProject accession number PRJNA1107029.
